# Tuberculosis knowledge, attitudes and practices of patients at primary health care facilities in a South African metropolitan: research towards improved health education

**DOI:** 10.1186/s12889-017-4825-3

**Published:** 2017-10-10

**Authors:** N. Gladys Kigozi, J. Christo Heunis, Michelle C. Engelbrecht, André P. Janse van Rensburg, H. C. J. Dingie van Rensburg

**Affiliations:** Centre for Health Systems Research & Development, University of the Free State, P.O. Box 399, Bloemfontein, 9300 South Africa

**Keywords:** Tuberculosis, Knowledge, Attitudes, Infection control practices, Free State Province, South Africa

## Abstract

**Background:**

Health education is important to empower patients and encourage their contribution towards tuberculosis (TB) control. In South Africa, health education activities are integrated into services provided at the primary health care (PHC) level. This study was conducted in a high TB burden metropolitan area in South Africa. The objective was to assess TB-related knowledge, attitudes and infection control practices of patients attending PHC facilities.

**Methods:**

In September and October 2015, a cross-sectional survey using fieldworker-administered questionnaires was conducted among patients older than 17 years attending 40 PHC facilities in the Mangaung Metropolitan. Convenience sampling was used to select patients. Participation in the study was voluntary. Descriptive, inferential and multivariate logistic regression analyses were performed. Statistical significance was considered at alpha <0.05 and 95% confidence interval.

**Results:**

A total of 507 patients’ data were included in the analysis. Most of the patients knew that TB transmission is facilitated by crowded conditions (84.6%) and that pulmonary TB is contagious (73.0%). Surprisingly, the majority of patients also believed that one can get TB from sharing toothbrushes (85.0%) or kissing (65.0%). An overwhelming majority of patients perceived TB to be serious (89.7%), and concurred that taking treatment (97.2%) and opening windows to prevent transmission in PHC facilities (97.0%) are important. Being employed (AOR: 11.5; CI: 4.8–27.6), having received TB infection control information from a PHC facility (AOR: 2.2; CI: 1.5–3.4), and being a TB patient (AOR: 1.6; CI: 1.02–2.46) increased the likelihood of adopting good infection control practices.

**Conclusion:**

These findings highlight the need for health education efforts to strengthen accurate information dissemination to promote sound TB knowledge and attitudes among patients attending PHC facilities. Health education efforts should also capitalise on the positive finding of this study that information dissemination at PHC facilities increases good infection control practices.

## Background

Despite recent World Health Organization (WHO) reports about a declining global trend, the burden of tuberculosis (TB) in South Africa remains unacceptably high [1, 2]. The high incidence of active TB infection, high proportion of latent infection and human immunodeficiency virus (HIV) comorbidity significantly undermine effective TB control in the country [3–5]. In 2014, out of the 318,193 TB cases notified approximately 61% were HIV co-infected and an estimated 24,000 HIV-negative and 72,000 HIV-positive TB cases died [1]. Health system failings in delivering adequate TB care have also exacerbated TB control challenges in the country [6]. For instance, drug-resistant forms of TB have spiralled largely as a result of late detection, poor treatment and management, and failure to retain TB patients on treatment [7–9]. Consequently, in 2014, approximately 9% of 218,231 laboratory-tested cases were confirmed to have drug-resistant TB [1].

Besides the high disease burden and systemic limitations, patients themselves may undermine TB control efforts through poor or high-risk infection control practices or non-adherence to treatment [10–12]. Poor/unhelpful patient conduct, negligence and resistance to participation in appropriate TB control efforts are motivated by individual, socio-economic and structural factors [11–14]. Negative behaviours and attitudes can be the direct result of patients’ own choices or the indirect result of a lack of knowledge [13]. Thus, empowering patients with appropriate and correct information and engendering positive attitudes towards TB and its curability is critical for the effective control of the disease. There is increasing recognition among researchers, health workers, and policy makers that encouraging patients to play a more active role in their health care improves the quality and efficiency of health care, and ultimately population-level health outcomes [15–19].

Health education is an important tool to foster patient empowerment and encourage their contribution towards TB control. In a study conducted in KwaZulu-Natal, TB-HIV co-infected patients found it easier to adhere to anti-retroviral treatment than anti-TB treatment, citing limitations such as poor communication, low patient involvement, and poor provider supervision of treatment by the TB programme [6]. Contrarily, the success of the HIV programme has been associated with commitment to provide adequate health education, treatment literacy and support to patients [6, 19].

In South Africa, health education activities are integrated into services provided at the PHC level. Health care workers are responsible for health education, information dissemination, and supporting patients through face-to-face consultation, health education campaigns, social mobilisation for different health programmes, and distribution of health education materials and media [20–22]. At most PHC facilities, due to lack of space, health education is conducted in large group lecture sessions in the waiting area facilitated by dedicated health educators or nurses [20]. Although these strategies may broaden health education reach, their actual impact on patient knowledge retention and attitudes and practices is generally not well established in the current setting. Previous research on patients’ TB-related knowledge, attitudes and practices revealed significant deficits in their levels of awareness regarding symptoms, transmission, prevention, and treatment of TB among various communities. The lack of TB awareness was shown to impact negatively on individual health-seeking behaviour, attitudes towards TB and infection control practices [11, 19, 23–30]. This study was conducted in a high burden metropolitan district in the Free State Province to assess the TB-related knowledge and attitudes, and infection control practices of patients attending PHC facilities. Findings provide information to guide TB control programme managers and PHC facility-based health care workers towards improving important aspects of TB-related health education.

## Methods

### Setting and design

In September and October 2015, a cross-sectional survey using fieldworker-administered questionnaires was conducted among patients older than 17 years attending health services across 40 PHC facilities in the Mangaung Metropolitan in the Free State. This Metropolitan, one of seven in South Africa, had a population of approximately 783,580 in 2015 [31]. The Metropolitan was purposively chosen, based on high recorded TB patient numbers and concurrent poor treatment outcomes. In 2013, TB incidence was estimated at 762 per 100,000 population and in 2012, the cure rate among smear positive cases was estimated at 68.8%, far below the national target of 82% and international target of 85% [31]. In 2012, HIV prevalence among public sector antenatal clinic clients was 30.3% in the Metropolitan, compared to an average of 29.5% in the country [32]. The Metropolitan comprises three sub districts: one city/central urban area and two small towns.

### Population, sampling and recruitment

The study population was defined as all patients (including TB patients) older than 17 years attending services across the 40 PHC facilities in Mangaung. During the second quarter of 2014, a total of 13,458 patients had accessed PHC services in the Metropolitan. From this number it was estimated that a minimum sample size of 374 patients was required for a margin of error of 5% and a confidence interval (CI) of 95%. Convenience sampling was used to select patients and in a bid to increase generalisability of findings patients were oversampled.

The study considered TB patients—those who were registered and were seeking TB services on the day of the field visit; and general patients—those who did not have TB and were seeking non-TB related services on the day of the field visit. The probability proportional-to-size technique was used to select patients from every facility. Due to logistical reasons, each facility was visited once and only those patients attending the facility on the day of the field visit were approached.

Patients were recruited for the study if they were older than 17 years and were able to express themselves verbally in either English or the predominant local language, Sesotho. Participation in the study was voluntary. Written informed consent was obtained prior to enrolling participants in the study. Patients were directed by a nurse to a recruiter and trained fieldworkers situated in a private room within the clinic. The fieldworkers conducted face-to-face interviews with the selected patients in English or Sesotho. In all instances, the patients who were approached agreed to participate in the study.

### Instrument design and measures

The fieldworker-administrated questionnaire was designed by selecting relevant questions from previous published research [11, 25, 27, 32–35]. The questionnaire comprised of four sections including: 1) socio-demographic information, 2) knowledge about TB, 3) attitudes towards TB, and 4) infection control practices. Information was obtained on patients’ socio-demographic characteristics such as age, gender (male or female), formal education attainment (primary school or lower, secondary school or National Senior Certificate and higher), employment status (employed or unemployed) and the location where patients attended PHC services (small town or city/central urban area). Nine items were used to measure patients’ knowledge about TB aetiology, transmission, susceptibility, and treatment. Patients were required to indicate their response to each question by choosing either ‘yes’, ‘no’ or ‘uncertain’. Patients’ attitudes were measured using 15 items. Patients were required to indicate their risk perception, views regarding medication, perceptions of care and treatment, health locus of control, concerns about other patients knowing their TB status and views about infection control at PHC facilities, by choosing from a 5-point Likert scale (1 = strongly agree, 2 = agree, 3 = uncertain, 4 = disagree and 5 = strongly disagree). In terms of infection control practices, patients were required to choose between ‘yes’ and ‘no’ for each of the nine items measuring infection control practices. They were also asked to indicate whether they had received information about TB infection control from PHC facilities (‘yes’ or ‘no’). The questionnaire was pilot tested at two PHC facilities outside the study setting for practicality and adjusted before being implemented for the target patient groups.

### Data analysis

Data were processed and analysed using SPSS (version 23) and described using frequency counts and percentages. The statistical significance of proportional differences were tested using chi-square tests and t-tests. With respect to TB knowledge, a score of one was allocated to every correct response and zero to every incorrect/‘uncertain’ response. A total knowledge score was also computed. Regarding attitudes, some items were reverse coded before frequencies and percentages were calculated. In terms of infection control-related practices, a score of one was allocated to the response ‘yes’ and zero to the response ‘no’. Total scores were then computed, with scores equal to or lower than the median denoting poor TB infection control practices and scores higher than the median indicating good practices.

Multiple logistic regression analysis was conducted to predict patients’ good TB infection control practices taking the following independent variables into consideration: gender, age, education, employment, geographic location, patient type, attitudes towards TB, and whether infection control information was received from the PHC facility. The outcome variable was ‘good TB infection control practices’, defined as total TB infection control practice scores higher than the median score. These scores were compared with ‘poor TB infection control practices’ (i.e. total scores below or equal to the median score). The assumptions of linearity, independence of errors, homoscedasticity, influential points and normality of residuals were met for the multiple logistic regression analysis. Univariate and adjusted odds ratios (ORs) and their corresponding CIs were calculated and significance was considered at alpha <0.05 and 95% CI.

## Results

### Socio-demographic characteristics

A total of 510 patients participated in the study, but data for three patients were eliminated from analysis due to missing demographic information. Table 1 shows that almost two-thirds (63.7%) of the patients were female. The mean age was 39.2 (standard deviation [SD]: 12.5) years, with more than half (56.4%) of the patients aged between 18 and 40 years. Two in every five patients had attained at least secondary school education (42.8%). Three in every four patients were unemployed (75.9%). The majority of patients were attending PHC facilities in city/central urban areas (71.0%).

**Table 1:** Patients’ socio-demographic characteristics (*N* = 507)

Variables	*n* (%)
Gender	
Female	323 (63.7)
Male	184 (36.3)
Age group^a^	
18–30	142 (28.0)
31–40	143 (28.2)
41–50	125 (24.7)
≥ 51	97 (19.1)
Education	
Primary or no school	153 (30.2)
Secondary school	217 (42.8)
National Senior Certificate or higher	137 (27.0)
Employment	
Unemployed	385 (75.9)
Employed	122 (24.1)
Location	
Small towns	147 (29.0)
City/central urban area	360 (71.0)

^a^Mean age = 39.2 (SD: 12.5) years

### Knowledge about TB

Patients’ knowledge about TB aetiology, transmission, susceptibility, and treatment is presented in Table 2. The mean TB knowledge score was 6.5 (SD: 1.3) out of 9. Patients’ knowledge concerning the cause of TB and key routes of transmission was poor; about two in every five (39.8%) patients did not know that TB was caused by a *bacillus*. Only one sixth of patients (15.0%) knew that TB cannot be transmitted through sharing toothbrushes and only about one-third (35.5%) were aware that TB cannot be transmitted through kissing.

**Table 2:** Patients’ knowledge of TB (*N* = 507)

Item	Correct response	
	*n*	%
Aetiology		
TB is caused by a germ (bacillus) (yes)	305	60.2
Transmission		
One can get TB from crowded conditions (yes)	429	84.6
One can get TB by sharing toothbrushes (no)	76	15.0
One can get TB through kissing (no)	180	35.5
One can get TB from a stranger more than from family (no)	227	44.8
Susceptibility		
Pulmonary (lung) is TB contagious (yes)	370	73.0
HIV positive persons are more likely to get TB (yes)	463	91.3
Treatment		
Most TB can be cured with medication (yes)	488	96.3
Susceptible TB treatment can take six months (yes)	474	93.7

Mean TB knowledge score = 6.5 (SD: 1.3)

While the majority of the patients knew that TB transmission is facilitated by crowded conditions (84.6%) and that pulmonary TB is contagious (73.0%), more than half (55.2%) of patients incorrectly associated TB transmission with strangers as compared to family members. Their understanding of susceptibility to TB was good; more than 90.0% knew that HIV positive people were at increased risk for TB. Likewise, a large majority (>90.0%) knew that TB was curable. Overall, gender (*p* = 0.168), education (*p* = 0.215), type of patient (*p* = 0.241) and location of PHC facility (*p* = 0.066) did not have a significant influence on patients’ TB knowledge.

### Patients’ attitudes towards TB

More than half (52.8%) of the patients strongly agreed that TB is a severe disease and two in every five patients (40.1%) strongly agreed that infection control at the PHC facilities was important. Six in every 10 patients (60.7%) strongly felt that treatment is important and more than half (54.7%) of the patients disagreed that they were likely to miss taking their medication. However, more than half (58.6%) of the patients were uncertain whether BCG vaccination prevents TB disease. Almost one-third of patients disagreed (32.4%) that TB disease can be avoided and just over half (58.1%) acknowledged that nurses are vital in administering TB medication. Almost half (49.3%) of the patients strongly disagreed that they would be embarrassed if other patients knew their TB status. Moreover, most patients (>50.0%) deemed infection control measures at the PHC facilities to be acceptable (Table 3).

**Table 3:** Patients’ attitudes towards TB (*N* = 507)

Item	Strongly agree	Agree	Uncertain	Disagree	Strongly disagree
	*n* (%)	*n* (%)	*n* (%)	*n* (%)	*n* (%)
Risk perception					
TB is a severe disease^a,b^	267 (52.8)	190 (37.5)	9 (1.8)	21 (4.2)	19 (3.7)
TB infection control precautions at clinic are important^a,b^	203 (40.1)	282 (55.7)	13 (2.6)	6 (1.2)	2 (0.4)
Open windows (even when it is cold) to prevent TB transmission is necessary in clinics^a,b^	327 (64.6)	164 (32.4)	8 (1.6)	5 (1.0)	2 (0.4)
Intentions					
When/if on TB treatment, I am likely to miss taking some medication^b^	8 (1.6)	63 (12.4)	6 (1.2)	276 (54.7)	152 (30.1)
Only very serious circumstances would prevent me from taking TB medication^b^	12 (2.4)	61 (12.1)	5 (1.0)	276 (54.7)	151 (29.8)
Care and treatment					
Taking TB medication as prescribed is important^a^	308 (60.7)	185 (36.5)	2 (0.4)	7 (1.4)	5 (1.0)
Clinic appointments are more trouble than worth^b^	12 (2.4)	28 (5.5)	1 (0.2)	310 (61.5)	153 (30.4)
Taking TB medication is/would be a lot of trouble	6 (1.2)	11 (2.2)	6 (1.2)	323 (64.0)	159 (31.5)
BCG vaccination prevents TB disease^b^	22 (4.4)	163 (32.4)	295 (58.6)	15 (3.0)	8 (1.6)
Health locus of control					
Getting TB disease can be avoided^a,b^	70 (13.8)	225 (44.5)	69 (13.6)	77 (15.2)	65 (12.5)
I (would) know better than the nurse when to stop taking TB medication^b^	7 (1.4)	32 (6.3)	9 (1.8)	294 (58.1)	164 (32.4)
Disclosure of TB status					
I am/will be embarrassed to have my TB status known by other patients at this clinic	4 (0.8)	15 (3.0)	1 (0.2)	237 (46.7)	250 (49.3)
Acceptability					
TB infection control precautions at this clinic are acceptable to me^a,b^	144 (29.0)	307 (61.8)	22 (4.4)	16 (3.2)	8 (1.6)
Measures to isolate TB patients at this clinic are acceptable to me^a,b^	79 (15.8)	292 (58.5)	13 (2.6)	87 (17.4)	28 (5.6)
Measures to isolate TB suspects at this clinic are acceptable to me^a,b^	76 (15.3)	277 (55.9)	16 (3.2)	92 (18.5)	35 (7.1)

^a^Item reverse coded during analysis (strongly agree = 5, agree = 4, uncertain = 3, disagree = 2 and strongly disagree = 1); ^b^Total less due to missing values

### Patients’ self-reported infection control practices

Table 4 indicates patients’ self-reported TB good infection control practices stratified by socio-demographic variables and factors influencing good practices. Just over two-thirds (68.4%) of patients reported good TB infection control practices, such as covering the mouth and nose with tissues when sneezing, disposing of used tissues in waste bins or washing of hands after contact with respiratory secretions, whether at home, at work, or in the PHC facility.

**Table 4:** Factors associated with patients’ good TB infection control practices

Item	Good infection control practices *n* = 347	Univariate OR (95% CI)	Adjusted OR (CI)
	*n* (row %)		
Gender			
Female (ref)	225 (69.7)	1	1
Male	122 (66.3)	0.9 (0.6–1.3)	0.7 (0.5–1.1)
Age			
18–30 (ref)	95 (66.9)	1	1
31–40	102 (71.3)	1.2 (0.7–2.0)	1.2 (0.7–2.0)
41–50	97 (77.6)	1.7 (1.0–3.0)	1.6 (0.8–3.0)
≥ 51	53 (54.6)	0.6 (0.4–1.0)	0.7 (0.4–1.5)
Education			
Primary or lower (ref)	98 (64.1)	1	1
Secondary school	148 (68.2)	1.2 (0.8–1.8)	0.9 (0.5–1.6)
National Senior Certificate or higher	101 (73.7)	1.6 (0.9–2.6)	0.9 (0.5–1.8)
Employed			
No (ref)	231 (60.0)	1	1
Yes	116 (95.1)	13.0 (5.5–30.0)	11.5 (4.8–27.6)
Location			
Small towns (ref)	94 (63.9)	1	1
City/central urban area	253 (70.3)	0.8 (0.9–2.0)	1.2 (0.7–1.8)
Patient type			
General (ref)	195 (66.6)	1	1
TB patient	152 (71.0)	1.2 (0.8–1.8)	1.6 (1.02–2.46)
TB attitudes (mean; SD)^a^	54.9 (5.2)	0.9 (0.94–0.97)	0.9 (0.92–0.99)
Received information about TB infection control from the PHC facility			
No (ref)	118 (58.7)	1	1
Yes	229 (74.8)	2.1 (1.4–3.1)	2.2 (1.5–3.4)

^a^Based on 14 items; Cronbach Alpha = 0.713

Results of univariate logistic regression analysis identified three factors that each had an independent and statistically significant influence on good infection control practices. These are the patients’ employment status, their attitudes towards TB, and whether they had received information about TB infection control at the PHC facilities. A multivariate logistic regression was then run to ascertain the factors influencing patients’ infection control practices after controlling for all variables in the model (i.e. gender, age, education, employment, location, patient type, attitudes towards TB, whether TB infection control was received from the PHC facility). A test of the full model against a constant only model was statistically significant, indicating that the predictors, as a set, reliably distinguished between poor and good infection control practices (chi-square = 97.8, *p* < 0.001, df = 11). The model explained 24.7% (Nagelkerke’s R^2^) of the variance in patients’ TB infection control practices. The overall prediction success was 71.1% with specificity (poor practices) of 37.5% and sensitivity (good practices) of 87.6%. The positive predictive value was 75.1% and the negative predictive value was 58.2%.

Results show that employment, whether patients received TB infection control information at PHC facilities, and patient type, made a statistically significant positive contribution to the prediction of patients’ good infection control practices after controlling for other factors in the model. Compared with their unemployed counterparts, those who were employed were 11.5 times (CI: 4.8–27.6) more likely to assume good infection control practices. Likewise, patients who had received information on infection control from PHC facilities were more than twice (AOR: 2.2; CI: 1.5–3.4) as likely to adopt good infection control compared to those who had not and TB patients were 1.6 (CI: 1.02–2.46) times more likely to assume good infection control practices than general patients. On the contrary, there was an inverse association between patients’ attitudes towards TB and good infection practices (AOR: 0.9; CI: 0.92–0.99).

## Discussion

This study assessed TB knowledge, attitudes and infection control practices of patients attending PHC facilities in the Mangaung Metropolitan of the Free State Province in South Africa. The results hold implications for health education at PHC facilities in this metropolitan and other similar settings. In terms of TB knowledge, the majority of patients in this study knew that pulmonary TB is transmissible and that crowded conditions and HIV infection increase the risk for TB infection. This is probably due to the heightened health system efforts to improve TB infection control in PHC facilities [36, 37]. As most PHC facilities are crowded [20], infection control activities such as triage and isolation of coughing patients, as well as adequate ventilation, are recommended to help reduce TB transmission [36].

However, in line with research in Ethiopia [27], it is surprising that a substantial proportion of patients in the current study incorrectly assumed that TB can also be transmitted through the sharing of toothbrushes or kissing. Reasons underlying this supposition are unclear but it could have resulted from patients having conflicting views about TB disease. From previous research in South Africa, patients described two types of TB where the first type was ascribed to cultural misconduct, while the second was described as ‘western’ TB – characterised by coughing, weight loss and night sweats. The patients explained that the latter type of TB was spread through being in close contact with sufferers as well as sharing food, drinks, cigarettes and eating utensils with sufferers [38]. Moreover, two in every five patients in this study were unaware that TB is caused by a *bacillus*. The finding corroborates earlier studies in South Africa where communities ascribed TB to other causes including witchcraft and sexual ‘misconduct’ [38, 39]. It is therefore essential for health workers to address these misconceptions and disseminate accurate information to patients, as ignorance may encourage stigmatisation and social isolation of those diagnosed with TB [18, 26, 27, 33, 34]. As far as TB treatment and prevention are concerned, a large majority of patients were aware that TB can be cured with appropriate medication and that treatment lasts at least 6 months, which can have a significantly positive impact on TB diagnosis and treatment adherence [26].

Patients in this study portrayed generally positive attitudes towards TB possibly due to their good knowledge of TB treatment. This may also be linked to the fact TB health education is provided to all patients attending PHC facilities regardless of their TB status. The positive attitudes may also be due to the presence of the fieldworkers, patients might have felt compelled to reflect positive attitudes. However, in line with two studies in Ethiopia [27, 34], patients in this study perceived TB to be a severe disease. Unlike the patients in Esmael and colleagues’ study [27] most patients in the current study were open to their TB status being known by other patients, probably because they did not anticipate discrimination. Previous research [34, 40] has demonstrated that negative attitudes towards TB are often underscored by the fear of being infected. Thus, health education efforts should reinforce information to counter fear of both contagion and TB stigma [40].

Results of the logistic regression showed that unemployed patients, those who did not receive information about TB infection control at PHC facilities, and general patients were less likely to adopt good infection control practices. This potentially signals a need to strengthen alternative channels to communicate health information outside of PHC facilities in a bid to increase responsiveness to health education. The unexpected finding that patients with positive attitudes towards TB were less likely to adopt good infection control practices necessitates further investigation. However, it is postulated that the lack of physical and financial resources, might have negatively impacted on patients’ ability to effectuate infection control appropriately. An assessment of infection control implementation in some PHC facilities in the KwaZulu-Natal Province of South Africa revealed impressive efforts by health workers to educate patients about TB and to disseminate information about cough etiquette. Nevertheless, masks and tissues for patients were unavailable at more than 80% of PHC facilities [41]. Systematic observations across the PHC facilities in the current study revealed that out of the 40 PHC facilities, only seven distributed tissues and only three distributed masks to coughing patients in waiting areas. At the same time, just more than three-quarters of the patients were unemployed and might have been unable to afford their own tissues and masks.

A limitation of this study is that the results are all based on self-reports by patients. Some patients might have felt obliged to present themselves in a positive light, thereby responding to questions in a manner that would be viewed favourably by others. Also, the convenient sampling of patients limits the generalisability of results. However, a strength of the study is that the results provide useful information for planning and improving health education interventions in Mangaung Metropolitan, and similar settings.

## Conclusion

In terms of knowledge, patients in this study demonstrated a good understanding of the contagiousness of and risk factors for TB, including HIV infection. However, some patients were unaware about the cause of TB and the key routes of its transmission. With respect to attitudes towards TB, most patients regarded TB to be serious and demonstrated positive attitudes towards treatment and care, as well as infection control at PHC facilities. Regarding infection control practices, most patients reported good infection control practices. Patients who had received information on TB infection control at PHC facilities were more likely to report good infection control practices. Socio-demographic factors also played a significant role in influencing good infection control practices. These findings highlight the need for health education efforts at PHC facilities in Mangaung and similar settings to address prevailing misconceptions about TB and to correct misinformation that might encourage social isolation of TB patients. Health education efforts should capitalise on the positive finding of this study, that information dissemination at PHC facilities increases good infection control practices.

## Abbreviations

HIV: Human Immunodeficiency Virus
PHC: Public health care
TB: Tuberculosis
WHO: World Health Organization
